# Photophysical Studies of a New Water Soluble Indocarbocyanine Dye Adsorbed onto Microcrystalline Cellulose and *β*-Cyclodextrin

**DOI:** 10.3390/molecules18055648

**Published:** 2013-05-15

**Authors:** Reda M. El-Shishtawy, Anabela S. Oliveira, Paulo Almeida, Diana P. Ferreira, David S. Conceição, Luis F. Vieira Ferreira

**Affiliations:** 1 UMTP - Unidade de Materiais Têxteis e Papeleiros e Departamento de Química, Universidade da Beira Interior, Rua Marquês d’Ávila e Bolama, Covilhã 6200-001, Portugal; E-Mails: elshishtawy@hotmail.com (R.M.S.); paulo.almeida@ubi.pt (P.A.); 2 Chemistry Department, Faculty of Science, King Abdulaziz University, P.O. Box 80203, Jeddah 21589, Saudi Arabia; 3 CQFM - Centro de Química - Física Molecular e IN - Instituto de Nanociência e Nanotecnologias, Instituto Superior Técnico, Universidade Técnica de Lisboa, Av. Rovisco Pais 1049-001, Lisboa, Portugal; E-Mails: asoliveira@ist.utl.pt (A.S.O.); diana.ferreira@ist.utl.pt (D.P.F.); david.conceicao@ist.utl.pt (D.S.C.); luisfilipevf@ist.utl.pt (L.F.V.F.); 4 C3i - Coordenação Interdisciplinar de Investigação e Inovação, Instituto Politécnico de Portalegre, P-7300-110, Portalegre, Portugal; 5 CICS - Centro de Investigação em Ciências da Saúde, Universidade da Beira Interior, Av. Infante D. Henrique, 6200-506 Covilhã, Portugal

**Keywords:** indocarbocyanine, microcrystalline cellulose, β-cyclodextrin, solid powdered samples, diffuse reflectance, laser induced luminescence, fluorescence quantum yields of powdered samples, lifetime distribution analysis

## Abstract

A water-soluble indocarbocyanine dye was synthesized and its photophysics were studied for the first time on two solid hosts, microcrystalline cellulose and β-cyclodextrin, as well as in homogeneous media. The inclusion of the indocarbocyanine moiety onto microcrystalline cellulose increased the dye aggregation with both H and J aggregates being formed. Adsorption on β-cyclodextrin enhanced aggregation in a similar way. The fluorescence quantum yields were determined for the powdered samples of the cyanine dye on the two hosts and a significant increase was observed relative to homogeneous solution. A remarkable concentration dependence was also detected in both cases. A lifetime distribution analysis has shown that the indocarbocyanine dye mainly occupies the amorphous part of cellulose and is not entrapped in the crystalline part of this host. In the β-CD case, the adsorption occurs outside the host cavity. In both hosts a strong concentration quenching effect is observed and only monomers emit. Both adsorptions may be explained by stereochemical constraints imposed by the two long sulphoethyl tails linked to nitrogen atoms of the indocarbocyanine dye.

## 1. Introduction

Polymethine cyanine dyes are among the most versatile functional dyes [1,2,3]. In addition to their use as traditional colorants [1,2,3], they have found potential applications in energy conversion [4,5,6], as laser dyes [1,2,7], as fluorescent labeling agents for biological applications [8,9,10] and in electro-optical applications [11,12,13,14,15,16,17]. Moreover, cyanine dyes, which were previously used as spectral sensitizers in photographic emulsions [18], are currently used as photo-initiators in photo-polymerization [19,20] and as potential sensitizers for photodynamic therapy [21,22,23,24,25].

Although cyanine dyes exhibit high extinction coefficients and efficient absorption over a long range of the optical spectrum [1,2,3,4,5,6], in fluid solution and at room temperature, the number of highly fluorescent dyes is rather small [26]. For sterically non-hindered cyanines in the monomeric form, this results from an efficient radiationless deactivation of the excited states by *trans-cis* isomerisation as the main decay route of the S_1_ state [1,2,26]. Therefore, the application of these compounds as potential fluorescent sensors [27] or as electro-, chemo-, and photoluminescent devices [28] is in most cases limited. Also, a major and often limiting problem in many fluorescence based diagnostic and imaging techniques is the photodegradation of the fluorophore [29]. Therefore, the knowledge of how to improve the fluorescence emission efficiency of cyanine dyes [30,31,32] and at the same time their photo-stability may be a valuable contribution towards the practical use of these dyes.

One of our group’s interests is the photophysical processes of several probes [30,31,32,33,34,35,36,37,38,39,40,41,42], including cyanine dyes [30,31,32,40,43] adsorbed on various heterogeneous powdered supports, particularly onto microcrystalline cellulose. Probes included within cyclodextrin [40,41] and calixarene [42] cavities were also investigated. Careful studies on the photophysics of several cyanine dyes adsorbed onto microcrystalline cellulose [30,31,32,43] showed that their deposition on this powdered substrate generally slows down the nonradiative deactivation processes that usually determine the photophysics of cyanine in solution [26] leading to an increase of the fluorescence quantum yields and lifetimes. As a result of the entrapment onto microcrystalline cellulose, the studied cyanine dyes revealed in some cases fluorescence quantum yields more than one order of magnitude [30,31,32,43] greater than those observed in solution for non-viscous solvents [26]. The same behaviour was observed by increasing the viscosity of the solvent [44], rigidifying the chemical structure of the cyanine dye molecule through its inclusion in rigid hosts as micelles and Langmuir-Blodgett films [45] or with the use of cyanines with a rigid chemical structure in solution [26]. Furthermore, fluorescent enhancement of cyanine dyes included within β-CD was also observed either for solution [46] or solid samples [40]. Either on microcrystalline cellulose or β-CDs phosphorescence enhancement was also observed [40]. Since inclusion on solid hosts globally improved the luminescence properties of cyanine dyes [30,31,32,43] it is expected that their photostability should follow the same pattern.

Microcrystalline cellulose is a very pure form of cellulose obtained by chemical treatment, where its amorphous regions are attacked and transformed into a highly crystalline residue [47]. It is constituted of glucose units joined by -1,4-glycosidic links [47] whose hydroxyl groups have a strong affinity for polar protic (alcohols) and aprotic (e.g., acetonitrile, acetone) solvents or probes that can reach them [34]. When a solution of the probe in one of these swelling solvents is added to microcrystalline cellulose, the cellulose to cellulose hydrogen bonds are replaced by cellulose to solvent bonds and the matrix swells to a degree which depends on the solvent used for sample preparation [34]. Probes can then penetrate into sub-microscopic pores of the solid substrate and stay strongly entrapped within the cellulose polymeric chains, after solvent removal [30,31,32,33,34,35,36,43].

Cyclodextrins (CDs) are water-soluble cyclic oligosaccharides consisting of six, seven and eight glucopyranose units (*α*, *β*, and γ-CD, respectively). All the glucose units are in a chair conformation, linked by *α*-(1,4) glycosidic oxygen bridges. This special arrangement gives a truncated cone-like structure with a central hydrophobic cavity. The inner diameters of *α*, *β*, and γ-CD are respectively 4.7–5.3, 6.0–6.5 and 7.5–8.3 Å [48]. The presence of the central hydrophobic cavity makes these molecules capable of forming inclusion complexes with many organic and inorganic guest molecules [48]. Among the three CDs, *β*-CD is readily available and has an appropriate cavity to accommodate organic molecules [40,41]. The restricted shape and size of the cavity constrains the guest molecule, therefore affecting its photochemical and photophysical properties [41]. The structural features of CDs also suggest that they might increase the solubility and bioavailability of the guest, as well as increase the photostability of the complexed cyanine dye included in their cavities.

Indocarbocyanine dyes, with different chain lengths of the central conjugated methine chain, are attractive mainly due to their ability to strongly absorb light from the blue to the near-infrared region [49]. Compared with other cyanine dyes, indocarbocyanine dyes have high solubility in solvents and have good thermal stability [50,51]. In fact, there is a lack of fluorophores with high fluorescence efficiency and good photostability that can be used as functional materials [52]. Another common disadvantage of cyanine dyes is their poor water solubility which is crucial to avoid strong dye aggregation. The inclusion of a sulphonate group (-SO_3_^−^ group) on the alkyl chain of cyanine dyes in known to improve dye solubility [53]. Such properties could therefore make indocarbocyanine dyes suitable candidates to be used as fluorescent probes for imaging techniques.

In this paper we report the synthesis of a novel water-soluble indocarbocyanine dye and its comparative photophysical behavior in two rigid matrices, namely microcrystalline cellulose and *β*-cyclodextrin. For this purpose, diffuse reflectance ground state absorption, laser induced luminescence and fluorescence lifetime determination of powdered solid samples were investigated.

## 2. Results and Discussion

### 2.1. Synthesis and Spectral Properties of 2-[3-(3,3-Dimethyl-1-(2-sulfoethyl)indolin-2-ylidene)prop-1-enyl]-3,3-dimethyl-1-(2-sulfoethyl)-3H-indolium *(**3**)* in Methanol

**Scheme 1:**
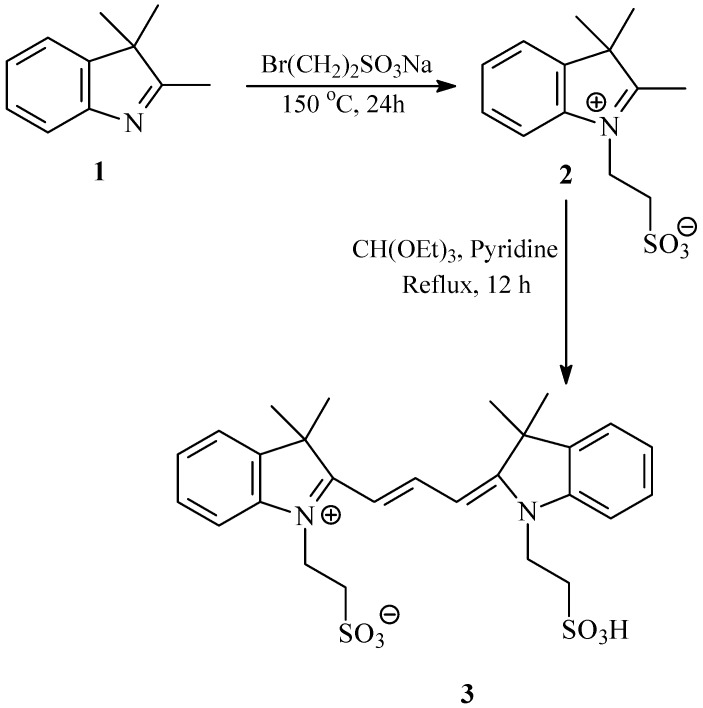
Synthesis of 2-[3-(3,3-dimethyl-1-(2-sulfoethyl)indolin-2-ylidene)prop-1-enyl]-3,3-dimethyl-1-(2-sulfoethyl)-3H-indolium, inner salt (**3**).

The water soluble dye **3** was synthesized by the two-step process depicted in Scheme 1, which involves *N*-alkylation with sodium bromoethane sulphonate followed by condensation with CH(OEt)_3_ in dry pyridine [54]. The structures of the new dye **3** was evidenced by its HRMS, ^1^H-NMR, ^13^C-NMR and IR data. It is worth noting that the new dye **3**, together with the reported pH sensitive fluorescent cyanine dye, were independently synthesized in one-pot process by a new Vilsmeier-type reaction [55].

### 2.2. UV-Vis Ground State Absorption of the Indocarbocyanine Dye in Solution

**Figure 1:**
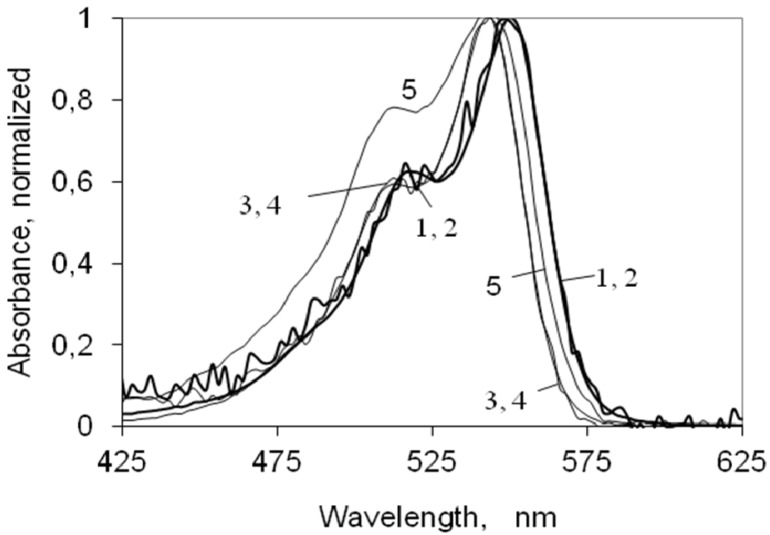
Absorption spectra of the 3,3'-di(2-sulfoethyl)-indocarbocyanine in ethanol (curves 1 and 2) and water (curves 3 to 5), normalized to the absorption maximum. The concentrations for the cyanine dye in ethanol are (1) 1x10^−7^ M (2) 1x10^−3 M; for the dye in water (3) 1 × 10−7^ M (4) 1 × 10^−4^ M and (5) 1 × 10^−3 M.^

Figure 1 presents the ground state absorption of the indocarbocyanine in ethanol and water at different concentrations. For each solvent, the spectra are normalized to unity at their absorption maximum. In ethanol the dye absorption has a peak at 550 nm with a vibrational shoulder at 516 nm (548 nm and 514 nm, respectively, in methanol); in water the absorption of the dye is about 6 nm shifted to the blue. Since the formation of the first singlet excited state is due to π- π transitions, one could expect that moving from ethanol to water a bathochromic shift should occur (due to the increase of the solvent polarity), however a deviation to the blue was observed in the absorption maxima, probably due to a stabilization of the + and − charges induced by water, from which a reduction of the resonance along the polymethine chain occurs.

Either in ethanol or water, this indocarbocyanine dye presents no significant aggregation for concentrations from 1 × 10^−7^ M up to 1 × 10^−4 M, as can be seen by the coincidence of the spectra over this range of concentrations. In water, for a concentration of 1 × 10−3 M, aggregate formation starts to be observed, as clearly indicated by the increase on the vibrational shoulder located at 512 nm, as described in literature for other cyanine dyes in water [30,31,32].^

### 2.3. UV-Vis Ground State Diffuse Reflectance of Powdered Solid Samples

#### 2.3.1. Indocarbocyanine Adsorbed on Microcrystalline Cellulose

**Figure 2:**
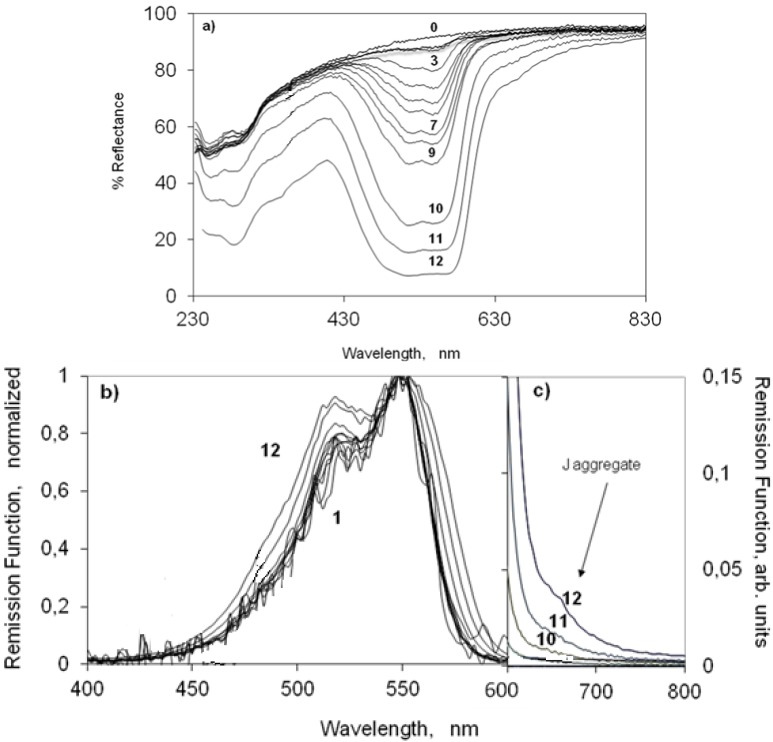
(**a**) Percentage Reflectance, (**b**) Remission function (normalized at the absorption maximum) and (**c**) Remission function of the 3,3'-di(2-sulfoethyl)-indocarbocyanine dye onto microcrystalline cellulose. The concentrations are (0) 0 (1) 0.001 (2) 0.01 (3) 0.05 (4) 0.1 (5) 0.2 (6) 0.3 (7) 0.5 (8) 0.75 (9) 1 (10) 5 (11) 10 (12) 25 μmol of the cyanine dye per gram of microcrystalline cellulose. Curve marked in bold in part b is from the dye in ethanol.

Figure 2 presents the ground state absorption of increasing loadings of the indocarbocyanine when adsorbed from ethanol on microcrystalline cellulose. Figure 2a shows the changes on the percentage reflectance as a function of the wavelength. The remission function of the same samples is presented in Figure 2b,c, normalized to the absorption maximum of the dye. For low concentrations of the dye deposited on microcrystalline cellulose, the indocarbocyanine absorbs from 400 to 650 nm with a maximum at 550 nm and a vibrational shoulder at 518 nm. This spectrum resembles very much the one we measured for the dye in ethanol (see Figure 1 or curve marked in bold in Figure 2b). This similarity makes it possible to ascribe the absorption of the low concentrated samples as the absorption of dye monomers on microcrystalline cellulose. The increase of the dye loading on cellulose above 0.01 μmol g^−1^ produces an increase in the absorption intensity at 518 nm, relatively to that of the monomer absorption maximum. This behaviour was extensively studied before for other cyanine dyes adsorbed on microcrystalline cellulose [30,31,32,43] and is due to the presence of H aggregates (sandwich dimers) of the dye. For concentrations higher than 1 μmol g^−1, a new band, not previously detected for this dye in water, appears at ~650 nm (see Figure 2a,c). Bands bathocromically shifted from monomer absorption that appear with the increase of concentration are characteristic of the absorption of J aggregates (head to tail aggregates) and have been observed for several cyanines either in solution [18] or on several substrates [18] including microcrystalline cellulose [30,31].^

#### 2.3.2. Indocarbocyanine on β-Cyclodextrin Solid Complexes

**Figure 3:**
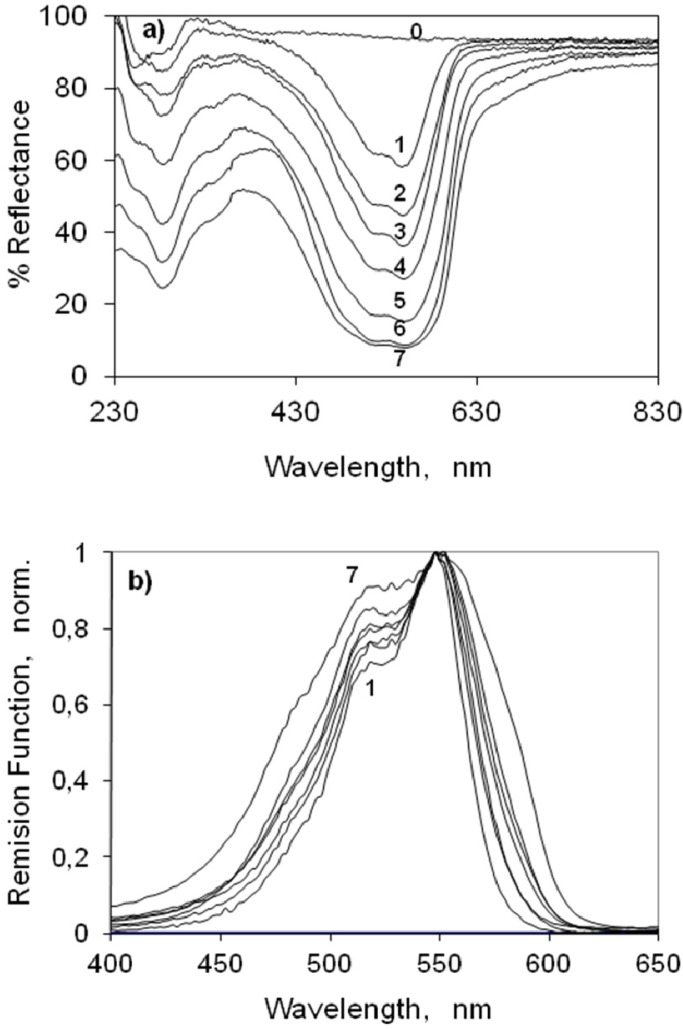
(**a**) Percentage Reflectance and (**b**) Remission function (normalized at the absorption maximum) of the 3,3'-di(2-sulfoethyl)-indocarbocyanine dye onto β-cyclodextrin. The molar ratios are (0) 0 (1) 1:1000 (2) 1:500 (3) 1:250 (4) 1:100 (5) 1:50 (6) 1:25 (7) 1:10 mol of the cyanine dye to moles of β-cyclodextrin.

**Figure 4:**
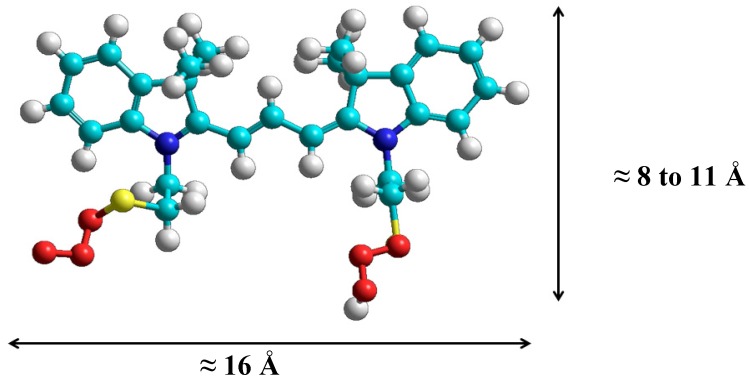
Structure of the 3,3'-di(2-sulfoethyl)-indocarbocyanine dye obtained with the use of HyperChem, version 6. The approximate dimensions of the trans isomer are indicated.

We also characterized the ground state absorption of the indocarbocyanine dye adsorbed onto β-cyclodextrin at several different molar ratios. The results obtained are presented in Figure 3. Monomers of the disulfoethyl indocarbocyanine on β-cyclodextrin peak about the same wavelength observed for microcrystalline cellulose and ethanol solution pointing also to adsorption onto polar sites of the β-CD. Observing curve 2 of Figure 4b, H dimers seem to be formed from very low molar ratios. The increase in the absorption at 518–520 nm indicates that they are certainly already present in the sample of 1:500 molar ratios. For the higher molar ratios of the indocarbocyanine/β-cyclodextrin solid complexes under study, *J* aggregates formation was also detected. In case of β-cyclodextrin, the absorption spectrum (curve 7 of Figure 3) is quite broad along with a weak shoulder as compared to other spectra. It seems that the band for J-aggregates is merged with the main band. J aggregates exist both in microcrystalline and β-cyclodextrin powdered solid supports in samples with high loadings of the cyanine dye. That is why the changes in the fluorescence spectra of the dye are almost similar in presence of both the hosts.

Although it is commonly mentioned in the literature that in solution, cyclodextrin addition usually inhibits the aggregation of cyanines and other dyes [45], this same increase on aggregate formation by cyanine’s adsorption onto β-cyclodextrin was also observed before by us for smaller cyanines within substrates which enabled dimer inclusion [40]. This result shows that the adsorption of this indocarbocyanine on the cyclodextrin strongly promotes aggregation, and that points to adsorption outside the β-cyclodextrin cavity and subsequent formation of H-dimers and J aggregates as shown next.

The dimensions of this indocarbocyanine were careful studied with the use of molecular modelling (HyperChem, version 6, Hypercube Inc. Scientific Software, Gainesville, FL, USA), see Figure 4. Comparing the dimensions of the dye with those of the β-cyclodextrin cavity (6.0–6.5 Å) we can easily conclude that the disulfoethyl indocarbocyanine cannot be included into this cyclodextrin cavity since the lowest dimension of the dye (presented in Figure 4) is in the 8–11 Å range, depending on the conformation of the adsorbed cyanine. The reported internal diameter for γ-cyclodextrin is about 8.5 Å. Therefore, even for this host, inclusion should probably be inhibited in most cases due to the long sulfoethyl tails of this cyanine. In any case, the initial idea of this study was to promote adsorption onto the surfaces of powdered solids and to study the eventual aggregation and not to separate the cyanine monomers by including them into cavities of the hosts.

### 2.4. Fluorescence Spectra and Fluorescence Quantum Yield

In order to evaluate whether the adsorption of the indocarbocyanine dye on the solid hosts selected for this work improved or not its fluorescence ability we further studied the emission of the dye in solution and on the two solid hosts.

#### 2.4.1. Indocarbocyanine in Solution

The fluorescence spectrum in ethanol is identical to the one measured in methanol and reported in the Photochem CAD database [56]. For ethanol, we determined a fluorescence quantum yield of 0.07 for this indocarbocyanine dye using the non-sulphonated dye as a reference compound (1,1'-diethyl-3,3,3',3'-tetramethylindocarbocyanine iodide).

#### 2.4.2. Indocarbocyanine on Microcrystalline Cellulose

**Figure 5:**
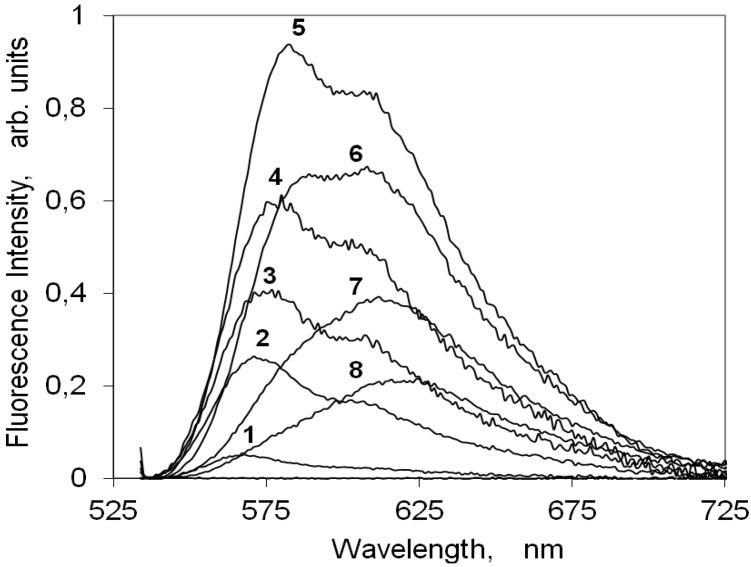
Steady-state emission spectra of the 3,3'-di(2-sulfoethyl)-indocarbocyanine dye on microcrystalline cellulose, excited at 525 nm. The sample concentration is (1) 0.01 (2) 0.05 (3) 0.1 (4) 0.5 (5) 1 (6) 5 (7) 10 (8) 25 μmoles of the cyanine dye per gram of microcrystalline cellulose.

Figure 5 presents the corrected fluorescence emission spectra of increasing loadings of the indocarbocyanine dye on cellulose. For this cyanine dye deposited on microcrystalline cellulose an intense fluorescence signal, peaking at about 580 nm with a vibrational shoulder at ~610 nm, was observed, contrarily to the low fluoresce quantum yield observed for non viscous solvents. The fluorescence intensity increases with dye loading for concentrations up to 1 μmolg^−1^. For higher concentrations a strong fluorescence quenching is observed, as is evident from Figure 5, by the intensity decrease of curves 6 to 8. A similar behavior was reported before for other families of cyanine dyes and attributed to formation of non-emissive H dimers.

**Figure 6:**
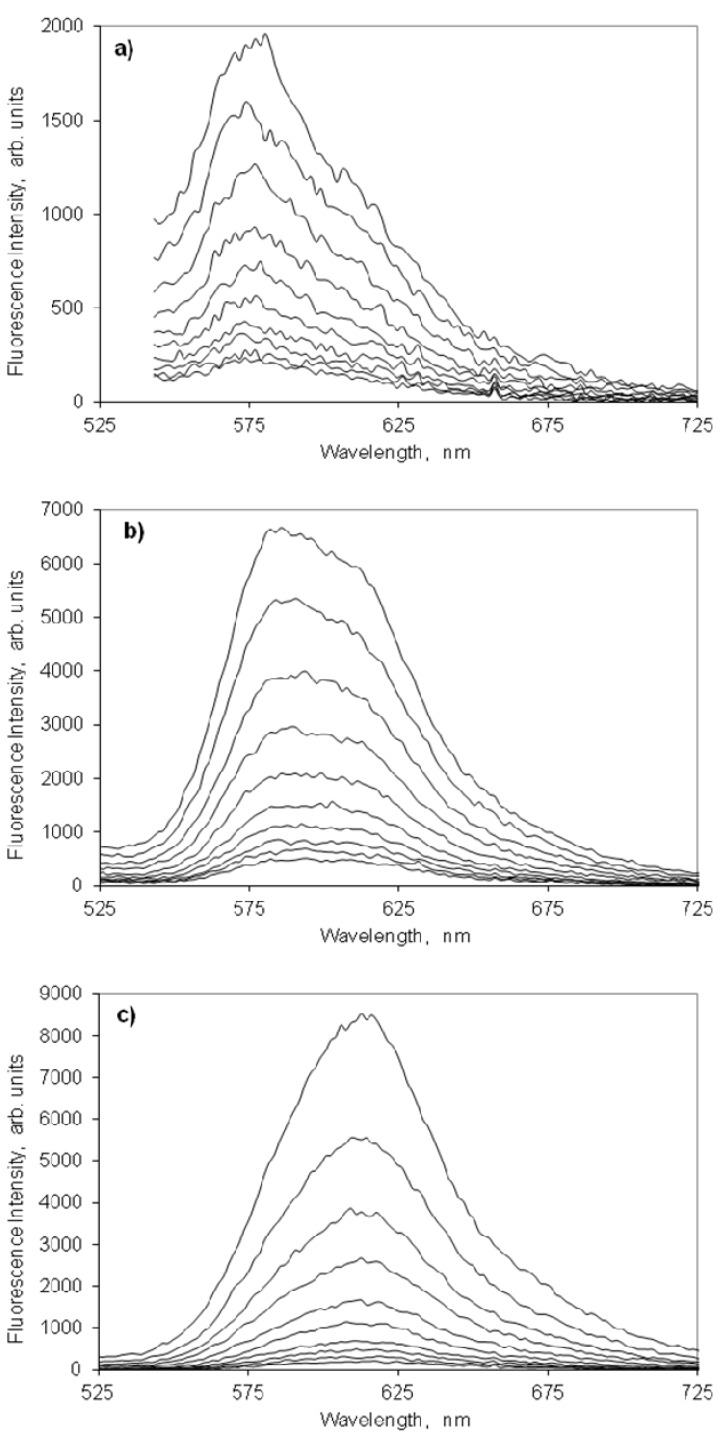
Laser induced time resolved spectra of the 3,3'-di(2-sulfoethyl)-indocarbocyanine dye on microcrystalline cellulose. Curve 1 = 0 ns; Step = 1 ns. Concentrations are (**a**) 0.05, (**b**) 0.5 and (**c**) 10 μmoles of the cyanine dye per gram of microcrystalline cellulose. The excitation wavelength was 337 nm.

Although the fluorescence emission maximum shifts from ~580 nm to ~620 nm with the increase on the dye loading on cellulose, this behaviour is not due to the presence of any other emitting species. In fact, the measurement of laser induced time resolved fluorescence spectra for several concentrations of this indocarbocyanine dye adsorbed on microcrystalline cellulose, presented in Figure 6, clearly shows that in the full range of investigated concentrations that is always the same species which is responsible for the fluorescence observed. From Figure 6 it is easily observed that in spite of the spectral shift of the fluorescence maximum, the lifetime of the process is always the same. The observed shift for the highest loadings originates from a strong reabsorption process due to the large overlap with ground state absorption. On microcrystalline cellulose the fluorescence lifetime of the monomers of this cyanine is about 2.4 ns.

**Figure 7:**
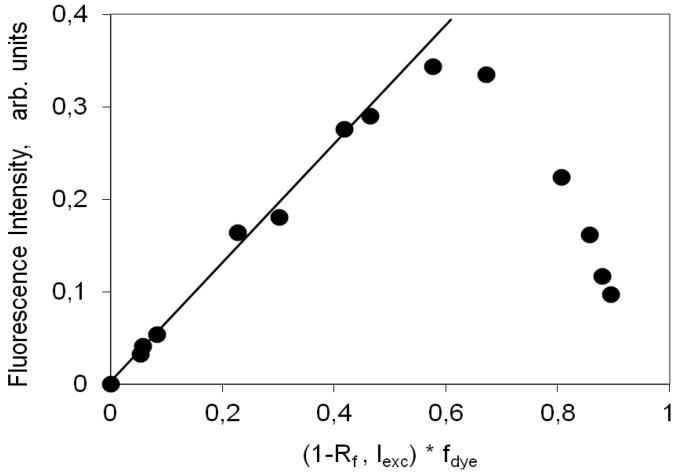
Variation of the intensity of fluorescence of the 3,3'-di(2-sulfoethyl)-indocarbocyanine dye onto microcrystalline cellulose measured as the total area under the corrected emission spectrum as a function of (1-R)f_dye_. The methodology used here was described in references [35,37].

Figure 7 presents the variation of the fluorescence intensity measured as the total area under the corrected emission spectrum as a function of (1-R)f_dye_ , a measure of the light absorbed by the dye at the excitation wavelength. When the fraction of light absorbed by the dye is small, there are only monomers absorbing and so I_F_ is linearly dependent on (1-R)f_dye_. For larger loadings non-fluorescent H aggregates are formed and as they absorb radiation they promote a strong decrease in I_F_. The monomer fluorescence curve, I_M_, does not continuously increase with concentration, C_0_, because although the concentration of monomers always increases, the fraction of light absorbed by them actually decreases as aggregates are formed. Since aggregates are non-fluorescent, the total fluorescence emission, I_F_, only reflects the I_M_ changes. The fluorescence quantum yield for this cyanine dye on microcrystalline cellulose is 65%. This high value shows that upon inclusion between cellulose chains this dye experiences an enormous increase on the rigidity of its chromophoric chain. This rigidity increase enabled to efficiently slow down the non-radiative deactivation processes responsible for the low fluorescence quantum yields usually observed in non-viscous solvents.

#### 2.4.3. Indocarbocyanine on β-Cyclodextrin Solid Complexes

**Figure 8:**
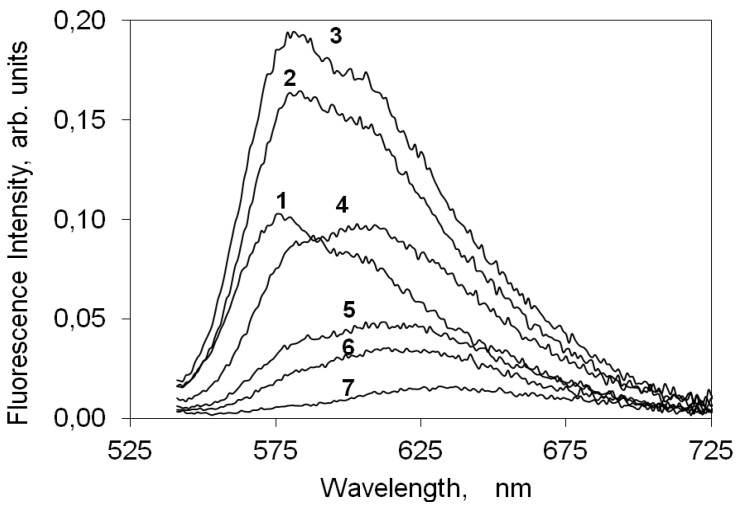
Steady-state emission spectra of the 3,3'-di(2-sulfoethyl)-indocarbocyanine dye onto β -cyclodextrin excited at 525 nm. The molar ratios are (0) 0 (1) 1:1000 (2) 1:500 (3) 1:250 (4) 1:100 (5) 1:50 (6) 1:25 (7) 1:10 of the cyanine dye to moles of β-cyclodextrin.

**Figure 9:**
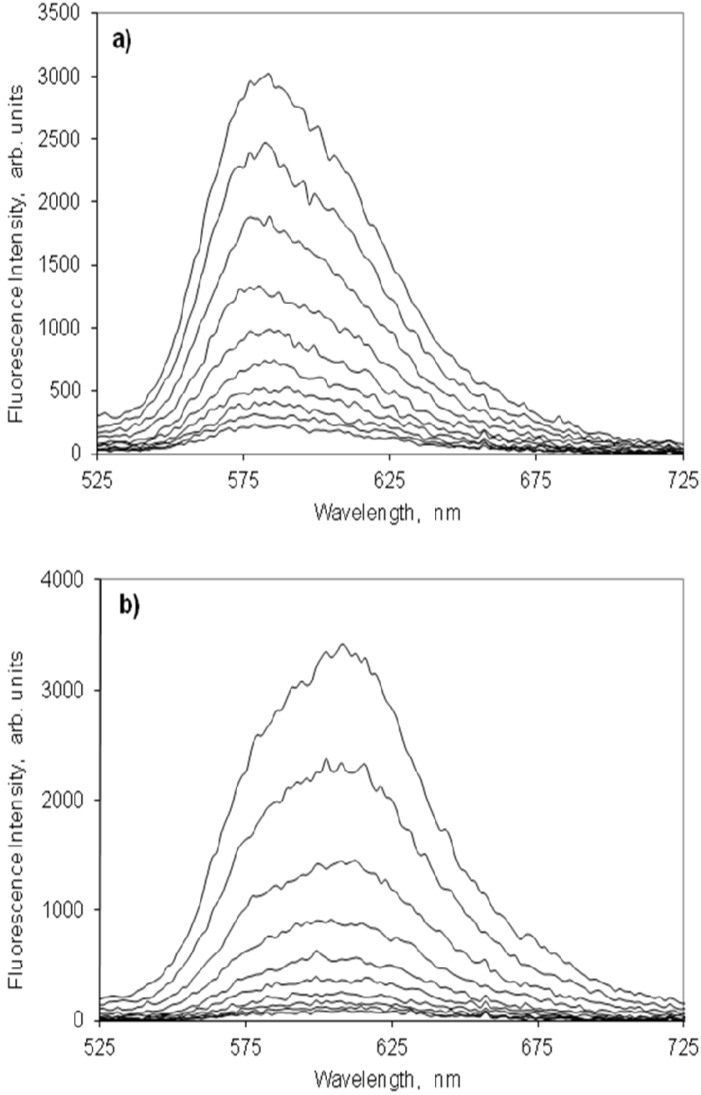
Laser induced time resolved spectra of the 3,3'-di(2-sulfoethyl)-indocarbocyanine dye onto β-cyclodextrin. Curve 1 = 0 ns; Step = 1 ns. Molar ratios are (**a**) 1:1000, (**b**) 1:50 moles of cyanine dye to moles of β-cyclodextrin. The excitation wavelength was 337 nm.

Figure 8 presents the fluorescence spectra of increasing molar ratios of the indocarbocyanine adsorbed onto β-cyclodextrin. As happened on microcrystalline cellulose, the fluorescence of this dye onto the β-CD is strong, peaks approximately at the same wavelengths and shows a similar concentration dependence. The maximum fluorescence intensity is observed for a molar ratio of 1:250 moles dye: β-cyclodextrin. Figure 9 shows the laser induced time resolved fluorescence spectra of low and high molar ratios of this dye on β-cyclodextrin, which also confirms the presence of a single emitting species in the full range of molar ratios investigated (slightly above 2 ns). Although ground state aggregates were found both on microcrystalline cellulose and on β-cyclodextrin (H and *J* aggregates), the fact that for both substrates a single fluorescence lifetime is measured irrespective the concentration of the samples proves the non emissive character of any of those aggregates.

For the disulfoethyl indocarbocyanine adsorbed on β-cyclodextrin the quantum yield was estimated to be about 38–50%. The higher uncertainty of the fluorescence quantum yield on this substrate compared to the value determined to microcrystalline cellulose aroused from the difficulty of preparing samples with molar ratios of dye:β-cyclodextrin lower than 1:1,000. Eventhough, it was possible to conclude that the fluorescence quantum yield of the dye adsorbed onto β-CD was 15–25% smaller than the one attained from the deposition of the same dye on microcrystalline.

This lower value observed with β-CD is not surprising, since the β-CD cavity does not have enough space to accommodate the molecules of this dye (Figure 4). Since the cavity has not sufficient space to accommodate one dye molecule, aggregation occurs outside the β-cyclodextrin cavity. 

### 2.5. Fluorescence Lifetime and Lifetime Distribution Analysis

The fluorescence lifetime for the indocarbocyanine in solution was calculated using the software provided by the EasyLife manufacturer; the determined value was 1.23 ns in ethanol.

**Figure 10:**
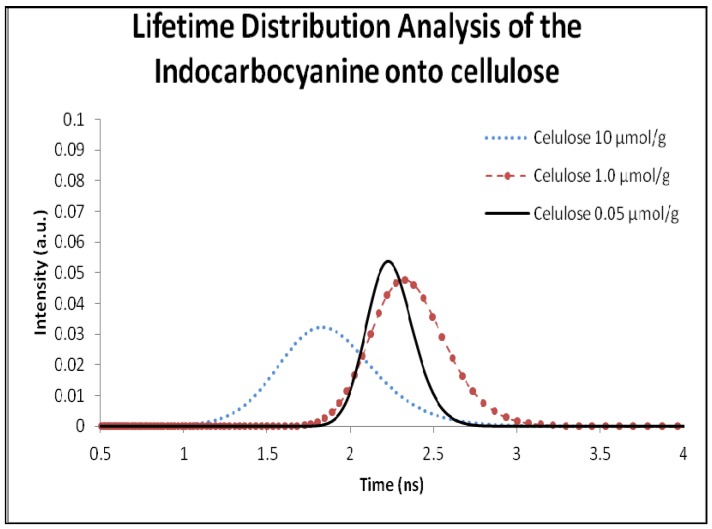
Lifetime distribution analysis of the 3,3'-di(2-sulfoethyl)-indocarbocyanine dye adsorbed onto microcrystalline cellulose. Samples concentration was 0.05, 1.0, 10.0 µmol of cyanine dye per gram of microcrystalline cellulose.

In previous studies, two fluorescence lifetimes were identified for powdered solid samples of the dyes eosin and phloxine on microcrystalline cellulose [39], accounting for the different locations of the dyes on the cellulosic environment: one very much ordered, where the dye is well entrapped into the crystalline cellulose polymer chains, and in that case, the dye exhibits the largest fluorescence lifetime due to the high constrain imposed by the entrapment, resulting in the decrease of the non-radiative pathways of deactivation namely the photoisomerization process. On the other hand, the distribution at shorter lifetimes is assigned to dyes located in more disorganized, more amorphous regions of cellulose. In this less constrained environment, adsorption sites are characterized by interactions with stereochemically available cellulose hydroxyl groups, enabling radiationless deactivation of the excited state namely through photoisomerization, leading to shorter emission lifetimes. Figure 10 shows some of the results obtained with the use of Lifetime Distribution Analysis (LDA) for the dye adsorbed onto microcrystalline cellulose.

The lifetime distribution analysis for the previously referred solid powdered samples revealed, in all cases, an increase of the lifetime value in comparison with the solution sample.

**Figure 11:**
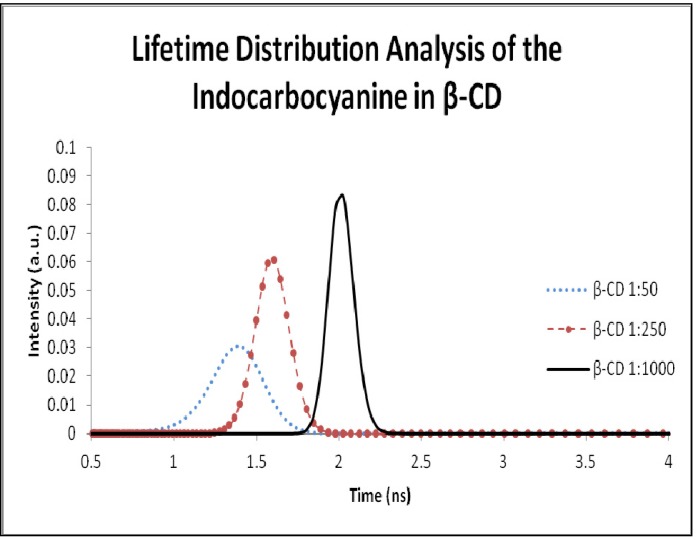
Lifetime distribution analysis of the 3,3'-di(2-sulfoethyl)-indocarbocyanine dye adsorbed onto β-cyclodextrin. Samples concentration was 1:1,000, 1:250 and 1:50 moles of cyanine dye per moles of β- cyclodextrin.

Figure 10 and Table 1 only show single distributions at shorter lifetimes indicating that all dye is located in the more amorphous regions of cellulose. The lifetime distribution analysis of the dye adsorbed into β-cyclodextrin is shown in Figure 11 and the correspondent lifetimes are presented in Table 1.

**Table 1:** Comparison between lifetime distribution analysis and mono-exponential analysis of the 3,3´-di(2-sulfoethyl)-indocarbocyanine dye adsorbed onto microcrystalline cellulose and on β-cyclodextrin.

Cellulose				β-Cyclodextrin			
Concentration	Fluorescence Lifetime (ns)	Χ^2^	MonoExp. Decay (ns)	Concentration	Fluorescence Lifetime (ns)	Χ^2^	MonoExp. Decay (ns)
10 µmol/g	1.8	0.78	2.0	1:50	1.4	0.65	1.6
1.0 µmol	2.3	0.65	2.5	1:250	1.7	0.73	2.1
0.5 µmol/g	2.4	0.51	2.7	1:500	2.3	0.93	2.6
0.05 µmol/g	2.2	0.74	2.4	1:1000	2.0	0.87	2.3

Considering the inner diameter of the β-CD (6.0–6.5 Å) and the size of the indocarbocyanine molecule, the inclusion the dye in the β-CD cavity is impossible. As we can see in the lifetime distribution analysis, the samples adsorbed into β-CD only exhibit one lifetime value, and considering that the dye doesn’t fit in the β-CD cavity, we assume that this value is from the dye outside the cavity. This result also corroborates our first interpretation based on the fluorescence lifetimes extracted from time resolved luminescence (about 2 ns for both hosts). For high loadings of the cyanine, and in what regards the displacement of the fluorescence emission maximum from 580 nm (low loadings of the dye, mostly monomers emitting) to about 620 nm (very high loadings of the dye), it may be due to fluorescence reabsorption rather then J aggregates emission, since the shape of the emission spectra for very high loadings of the cyanine reflects closely the J aggregate absorption, as can be observed in Figure 6 and Figure 9. In accordance, a severe concentration quenching effect was evidenced for both hosts slightly decreasing the observed lifetime, as data from Figure 10 and Figure 11 clearly show.

## 3. Experimental

### 3.1. Materials and Characterization

2,3,3-Trimethyl-3*H*-indolenine, sodium 2-bromoethanesulfonate, triethyl orthoformate, pyridine, chloroform, ethanol, methanol, diethyl ether, and chloroform of the highest purity available were purchased from Aldrich (St. Louis, MO, USA). Molecular sieves (3 and 4 Å, 4–8 mesh, Aldrich, previously activated by slow heating up to 250 °C under vacuum) were used to dry ethanol for microcrystalline cellulose sample preparation. Microcrystalline cellulose (Fluka DSO, Fluka, St. Louis, MO, USA) with 50 μm average particle size and *β*-cyclodextrin (Aldrich) were used as rigid matrices.

Synthesis of the dye (Figure 1) was monitored by TLC on aluminum plates pre-coated with Merck silica gel 60 F_254_ (0.25 mm) using chloroform/methanol (25%). ^1^H- and ^13^C-NMR spectra (250.13 and 62.9 MHz, respectively) were recorded on a Brüker AC 250P spectrometer, at room temperature, in DMSO-*d*_6_, with TMS as internal standard. Infrared spectra were performed on a Mattson 5000-FTS FTIR spectrometer. All samples were prepared by mixing FTIR-grade KBr (Aldrich) with 1% (w/w) dye, and grinding to a fine powder. Spectra were recorded over the 400–4000 cm^−1^ range without baseline corrections. High Resolution Fast Atom Bombardment Mass Spectra (HR FAB-MS) was measured on a Micromass AutoSpec M, operating at 70 eV, using a matrix of 3-nitrobenzyl alcohol, and melting point was determined in open capillary tubes in a Büchi 530 melting point apparatus and is uncorrected. Absorption and fluorescence spectra of methanolic solution of the dye were recorded on a Unicam HeλIOSα spectrophotometer and a SPEX FluoroMax 3109 spectrofluorophotometer, respectively.

### 3.2. Synthesis of 2-[3-(3,3-Dimethyl-1-(2-sulfoethyl)indolin-2-ylidene)prop-1-enyl]-3,3-dimethyl-1-(2-sulfoethyl)-3H-indolium, Inner Salt *(**3**)*

A mixture of 2,3,3-trimethyl-3*H*-indolenine (1.59 g, 10 mmol) and sodium 2-bromoethanesulfonate (2.11 g, 10 mmol) was melted at 150 °C with stirring for 24 h, and then cooled to room temperature. The thick material obtained was dissolved in methanol and precipitated with diethyl ether. The precipitate was filtered, washed with diethyl ether and dried under vacuum to afford a rose pink powder of salt **2**, which was used in the subsequent step without further purification. The product was mixed with triethyl orthoformate (3.5 mL, 21.07 mmol) and dry pyridine (15 mL) and refluxed with stirring for 12 h. The room temperature cooled mixture was mixed with diethyl ether and refrigerated. The resultant precipitate was filtered, washed with diethyl ether, and dried. Silica gel column chromatography of the product using a chloroform/methanol gradient (5–30%) as eluent affords the corresponding symmetric indocarbocyanine dye **3** as a dark red powder (2.85 g, 54% yield). M.p. > 300 °C. ^1^H-NMR (DMSO-*d_6_*) δ_ppm_: 1.68 (12 H, s, 3,3'-CH_3_), 2.96 (4H, m, NCH_2_CH_2_SO_3_H + NCH_2_CH_2_SO_3_), 4.35 (4H, m, NCH_2_CH_2_SO_3_H + NCH_2_CH_2_SO_3_), 6.52 (2H, d, *J* = 12.5 Hz, α-CH + α'-CH), 7.28 (2H, t, *J* = 7.5 Hz, 6&6'-CH_ar_), 7.37–7.44 (4H, m, 4,4' and 5,5'-CH_ar_), 7.62 (2H, d, *J* = 7.5 Hz, 7,7'-CH_ar_), 8.30 (1H, t, *J* = 12.5 Hz, β-CH). ^13^C-NMR (DMSO-*d_6_*) δ_ppm_: 27.36 (4C, 3,3'-CH_3_), 39.52 (2C, NCH_2_CH_2_SO_3_H + NCH_2_CH_2_SO_3_), 47.62 (4C, NCH_2_CH_2_SO_3_Na + NCH_2_CH_2_SO_3_), 48.97 (2C, 3,3'-C), 103.27 (2C, α-CH + α'-CH), 111.62 (2C, 7,7'-CH_ar_), 122.47 (2C, 4,4'-CH_ar_), 125.11 (2C, 5,5'-CH_ar_), 128.1 (2C, 6,6'-CH_ar_), 140.67 (2C, 3a,3a'-C), 141.89 (2C, 7a,7a'-C), 149. 69 (C, β-CH), 173.81 (2C, 2,2'-C). IR ν_max_/cm^−1^: 3414, 2946, 2875, 1561, 1433, 1354, 1206, 1146, 1040, 926, 750. UV-Vis (methanol): λ_max_ = 548 nm and ε = 68367 M^−1^ cm^−1^. HRMS (FAB, 3-NBA) calc. for C_27_H_33_N_2_O_6_S_2_: 545.1780; found: 545.1775. 

### 3.3. Sample Preparation

Microcrystalline cellulose was dried under vacuum (*ca.* 10^−3^ mbar) at 60 °C for at least 24 h before sample preparation. The samples were prepared using the solvent evaporation method [30,31,32,33,34,35,36,40]. This method consists of the addition of a solution containing the indocarbocyanine in dried ethanol to the previously dried microcrystalline cellulose, followed by slow solvent evaporation from the slurry under stirring in a fume cupboard. The final solvent removal was performed overnight under vacuum, at 40 °C, in an acrylic chamber with an electrically heated shelf (Heto, Model FD 1.0–110) with temperature control (25 ± 1 °C), at a pressure of *ca*. 10^−3^ Torr.

Solid complexes of the indocarbocyanine dye with β-cyclodextrin of several different molar ratios (1:1,000, 1:500, 1:250, 1:100, 1:50, 1:25 and 1:10 moles of indocarbocyanine dye to moles of β-cyclodextrin) were prepared by adding a water solution of the dye with the appropriate concentration to a saturated water solution of the β-cyclodextrin (*ca.* 10^−2^ M). After magnetic stirring for 48 hours, the samples were lyophilized (Heto, Model FD 1.0-110). The resulting solid complexes were washed with diethyl ether to remove any non-complexed dye [41]. Final solvent traces were removed under reduced pressure as described above.

### 3.4. Experimental Methods

#### 3.4.1. Ground-State Absorption in the UV-Visible

Ground-state absorption spectra of the dye/ microcrystalline cellulose and dye**/**β-cyclodextrin samples were measured on an OLIS 14 UV/Vis/NIR spectrophotometer with a diffuse reflectance attachment. The integrating sphere is 90 mm diameter, internally coated with a standard white coating. The standard apparatus was modified to include the possibility of using short-wave-pass filters which excludes the sample luminescence from reaching the detector (Hamamatsu, Model R955). Further experimental details and a full description of the system calibration used to obtain accurate reflectance, (R), measurements are given in reference [35,40]. 1 cm quartz cells were used for measurement of powdered samples. The remission function, F(R), of a probe adsorbed or included onto a solid powdered adsorbent and calculated with the use of the Kubelka-Munk equation for optically thick samples (F(R) = (1−R)^2^/2R ), after correction for the blank, is proportional to the host concentration [30,31,32,33,34,35,36,40]. Solution measurements were made using the same apparatus in the normal transmission mode. To register solution’s spectra quartz cell with 1 cm, 1 mm, 0,1 mm path length were used. 

#### 3.4.2. Laser Induced Fluorescence Emission

Corrected fluorescence emission spectra of the powdered samples were measured in the laser induced time resolved emission system, described in references [40,41,42]. The excitation source is the 337.1 nm pulse of a nitrogen laser (model PL2300, Photon Technology Instruments) that gives ~1.0 mJ/pulse and has a pulse width of 600 ps. The light emitted from the powdered samples was measured in a front surface arrangement by a gated intensified charged coupled device (ICCD, model i-Star 720, Andor). With this set-up, both fluorescence and phosphorescence spectra were easily available (by the use of the variable time gate width and start delay facilities of the ICCD). The determination of fluorescence quantum yield in solution and on solid supports was done in accordance with references [35,37].

#### 3.4.3. Fluorescence Lifetimes and Lifetime Distribution Analysis

Fluorescence lifetimes were determined using Easylife V™ equipment from OBB (lifetime range from 90–100 ps to 3 μs). This technique uses pulsed light sources from different LEDs (525 nm in this case) and measures fluorescence intensity at different time delays after the excitation pulse. In this case, 570 nm cut-off filter was used at emission. The instrument response function was measured using a Ludox scattering solution. FelixGX software from OBB was used for fitting and analysis of the decay dynamics, a lifetime distribution analysis, the exponential series method (ESM) and results were confirm by 1–4 exponentials method. In the exponential series method (ESM), the fluorescence decay is approximated by a sum of exponential series weighted by variable amplitudes. No specific profiles for the amplitudes determination are used here, and the amplitudes and lifetimes are obtained by minimizing the chi-square function where the excitation pulse profile and the instrumental response function are taken into account in the convolution matrix. 

Lifetime distributions analysis (LDA) for emissions of probes adsorbed onto heterogeneous surfaces [38] are very convenient methodologies to treat the phosphorescence or fluorescence decay data because these reflect the multiplicity of sites available for the probe molecules on the specific surface under study [38]. The use of a sum of several exponentials to analyse the decay of probes onto heterogeneous surfaces is a description without physical meaning [38]. The validation of the conclusions of the LDA analysis should be simultaneously sustained by other spectroscopic studies. 

In this work, and due to the quite short solution lifetime of the indocarbocyanine in solution or adsorbed, the software provided by the EasyLife manufacturer was used, which enables lifetime evaluation with accuracy, starting at about 90 ps, up to 3µs. The fluorescence decay is approximated by a sum of exponential series weighted by variable amplitudes. No specific profiles for the amplitudes determination are used here, and the amplitudes and lifetimes are obtained by minimizing the chi-square function where the excitation pulse profile and the instrumental response function are taken into account in the convolution matrix.

## 4. Conclusions

The wide application of indocarbocyanines as functional dyes in hi-tech applications has prompted the present work in order to gain some knowledge about how to increase the fluorescence emission efficiency and at the same time how to increase the photostability of these dyes. Consequently, a new water-soluble indocarbocyanine dye was synthesized and the adsorption of the dye at different concentrations onto microcrystalline cellulose and β-cyclodextrin, respectively, was foreseen as a convenient way to improve such properties. Thus the photochemistry of the indocarbocyanine was investigated in both hosts. The inclusion of the indocarbocyanine onto microcrystalline cellulose and β-cyclodextrin effectively increased the aggregation ability of this dye since both H and J aggregate formation was easily obtained by increasing the concentration of the dye on these hosts. When the indocarbocyanine dye is deposited onto microcrystalline cellulose from an ethanolic solution the swelling of the cellulose chains enables the penetration of the dye within the long chains of the substrate where its stays entrapped, after solvent removal, preferably in the amorphous region of this host. This location of the dye within the cellulose matrix seems to be suitable for sandwich and heat-to-tail arrangements of its various monomers leading to H and J aggregate formation, respectively. A lifetime distribution analysis study suggests that the indocarbocyanine dye manly occupies the amorphous part of cellulose instead of being entrapped into the crystalline part of this host. In the β-CD case, the adsorption occurs outside the host cavity, and evidences a strong concentration quenching effect. Both adsorptions may be explained by steriochemical constrains imposed by the two long sulphoethyl tails linked to nitrogen atoms of the indocarbocyanine dye. Even though fluorescence quantum yield determinations showed that this cyanine dye adsorbed onto these two powdered solid hosts still experiences a remarkable increase in fluorescence quantum yield relative to homogeneous solution, with a strong concentration dependence being detected in both hosts.
